# Severe nivolumab-induced pneumonitis preceding durable clinical remission in a patient with refractory, metastatic lung squamous cell cancer: a case report

**DOI:** 10.1186/s13045-017-0433-z

**Published:** 2017-02-28

**Authors:** Hong Li, Weijie Ma, Ken Y. Yoneda, Elizabeth H. Moore, Yanhong Zhang, Lee L. Q. Pu, Garrett M. Frampton, Michael Molmen, Philip J. Stephens, Tianhong Li

**Affiliations:** 1 Division of Hematology/Oncology, Department of Internal Medicine, University of California Davis School of Medicine, University of California Davis Comprehensive Cancer Center, 4501 X Street, Suite 3016, Sacramento, CA 95817 USA; 2 Department of Geriatrics, Peking University First Hospital, Beijing, China; 3 Division of Pulmonary, Critical Care, and Sleep Medicine, Department of Internal Medicine, University of California Davis School of Medicine, Sacramento, CA USA; 4 Department of Internal Medicine, Veterans Affairs Northern California Health Care System, Mather, CA USA; 5 Department of Radiology, University of California Davis School of Medicine, Sacramento, CA USA; 6 Department of Pathology and Laboratory Medicine, University of California Davis School of Medicine, Sacramento, CA USA; 7 Division of Plastic Surgery, Department of Surgery, University of California Davis School of Medicine, Sacramento, CA USA; 8 Foundation Medicine, Inc., Cambridge, MA USA

**Keywords:** PD-1 inhibitor, Nivolumab, Cancer immunotherapy, Immune-related adverse event, Pneumonitis, Complete remission, Tumor genomic profiling, Targeted exome sequencing, Tumor mutation burden, HLA

## Abstract

**Background:**

Programmed cell death 1 (PD-1) and its ligand 1 (PD-L1) inhibitors have quickly become standard of care for patients with advanced non-small cell lung cancer and increasing numbers of other cancer types. In this report, we discuss the clinical history, pathological evaluation, and genomic findings in a patient with metastatic lung squamous cell cancer (SCC) who developed severe nivolumab-induced pneumonitis preceding durable clinical remission after three doses of nivolumab.

**Case presentation:**

A patient with chemotherapy-refractory, metastatic lung SCC developed symptomatic pneumonitis by week 4 after nivolumab treatment, concurrently with onset of a potent antitumor response. Despite discontinuation of nivolumab after three doses and the use of high dose oral corticosteroids for grade 3 pneumonitis, continued tumor response to a complete remission by 3 months was evident by radiographic assessment. At the time of this submission, the patient has remained in clinical remission for 14 months. High PD-L1 expression by immunohistochemistry staining was seen in intra-alveolar macrophages and viable tumor cells in the pneumonitis and recurrent tumor specimens, respectively. Tumor genomic profiling by FoundationOne targeted exome sequencing revealed a very high tumor mutation burden (TMB) corresponding to 95–96 percentile in lung SCC, i.e., 87.4–91.0 and 82.9 mut/Mb, respectively, in pre- and post-nivolumab tumor specimens. Except for one, the 13 functional genomic alterations remained the same in the diagnostic, recurrent, and post-treatment, relapsed tumor specimens, suggesting that nivolumab reset the patient’s immune system against one or more preexisting tumor-associated antigens (TAAs). One potential TAA candidate is telomerase reverse transcriptase (TERT) in which an oncogenic promoter -146C>T mutation was detected. Human leukocyte antigen (HLA) typing revealed HLA-A*0201 homozygosity, which is the prevalent HLA class I allele that has been used to develop universal cancer vaccine targeting TERT-derived peptides.

**Conclusions:**

Nivolumab could quickly reset and sustain host immunity against preexisting TAA(s) in this chemotherapy-refractory lung SCC patient. Further mechanistic studies are needed to characterize the effective immune cells and define the HLA-restricted TAA(s) and the specific T cell receptor clones responsible for the potent antitumor effect, with the aim of developing precision immunotherapy with improved effectiveness and safety.

## Background

First generation monoclonal antibodies against programmed death receptor 1 (PD-1) and its ligand 1 (PD-L1) have quickly become standard of care in second-line and more recently first-line treatment over chemotherapy for patients with advanced non-small cell lung cancer (NSCLC) and an increasing number of other cancer types [1–6]. Although these drugs are generally better tolerated than chemotherapy, they are associated with unique and variable immune-related adverse events (irAEs) that if not recognized and treated promptly may increase morbidity and rarely cause mortality [7, 8]. We present a case of lung squamous cell cancer (SCC) with complex tumor genomic alterations to highlight the striking yet largely untapped potential of PD-1 inhibitor therapy, as well as the need for further clinical and translational research to optimize the safety and efficacy of cancer immunotherapy.

## Case presentation

A 67-year-old Caucasian man, former light smoker (4 pack-year, quit >45 years ago) with refractory metastatic lung SCC, received nivolumab as third line systemic therapy. He initially presented with SCC of unknown primary with a 5.5-cm mass in the right axilla and metastases in 4 of 28 regional lymph nodes. After complete surgical resection, the patient received adjuvant chemotherapy with gemcitabine and paclitaxel for 2 cycles, followed by adjuvant radiation to the right axilla and an additional 2 cycles of gemcitabine and paclitaxel uneventfully over a 7-month period. Review of the CT scan that was performed prior to adjuvant radiation to the right axilla noted a new 10-mm nodule in the right lower lobe (RLL) of the lung that had not been previously appreciated. In retrospect, the nodule was thought to be the primary lung cancer. It was no longer present on a subsequent scan after treatment. He tolerated systemic chemotherapy relatively well but had grade 1 radiation pneumonitis in the right upper lobe (RUL) of the lungs detected by fluorodeoxyglucose (FDG)-positron emission tomography (PET)-computed tomography (CT) scan at completion of radiation. Unfortunately, surveillance PET/CT scan 4 months after completion of his last dose of chemotherapy revealed a right axillary mass of 2.8 × 1.5 cm with maximum standardized uptake values (SUVmax) of 3.8, a RLL lung nodule which had undergone cavitation and growth, measuring 2.2 × 2.7 cm with SUVmax of 6.5, and a new 5-mm lung nodule in the left lower lobe (LLL). The radiation pneumonitis in the RUL had improved without any intervention. Biopsy of the recurrent right axillary mass and RLL lung mass confirmed recurrent, moderately differentiated SCC of most likely lung primary by immunohistochemistry stains (p40: positive and TTF1: negative). Given the patient was initially thought to have locally advanced SCC of unknown primary, all these tumor specimens were sent for comprehensive tumor genomic profiling by FoundationOne (Foundation Medicine, Cambridge, MA) (Table 1, first three columns). Available histopathological and radiographic data were reviewed at our institutional multidisciplinary thoracic oncology tumor board. Skin primary was not favored as cutaneous SCC rarely has distant metastasis and the patient did not have any skin lesion. Both the initial and recurrent tumors were present in the RLL of the lung. Together with the morphological and immunohistochemistry (IHC) evaluation, a diagnosis of lung SCC with RLL primary and metastases to the right axilla, mediastinum, and left lung was made.

**Table 1:** Summary of tumor genomic profiling by FoundationOne assay

Functional genomic alterations (date of specimen)	A: primary axillary tumor (11/19/2013)	B: recurrent axillary tumor (1/8/2015)	C: recurrent lung tumor (2/10/2015)	D: progressive lung tumor (9/29/2015)
	Mutation allele frequency (MAF) (%)			
TERT promoter -146C>T	6%	22%	35%	47%
PIK3CA Q661K	7%	24%	18%	23%
ARID2 R542^a^	6%	22%	27%	30%
CDC73 G28^a^		9%		
CDKN2A A100V	11%	40%	48%	47%
CDKN2A R58^a^	12%	39%	46%	49%
FANCC E539K	8%	21%	28%	29%
FAT1 N126fs^a^20	3%	22%	24%	24%
NOTCH1 splice site 5639-1G>A	18%	35%	40%	35%
PTCH1 P253L	3%	9%	15%	13%
SMAD4 Q180^a^	11%	25%	36%	37%
SPEN S346F	10%	26%	17%	16%
TP53 Q317^a^	2%	9%	10%	14%
TP53 Y163H	14%	37%	47%	44%
Number of functional genomic alterations	13	14	13	13
TMB (mut/Mb)	70.3	91.0	87.4	82.9
TMB percentile in lung SCC	98.8%	99.1%	99.1%	99.0%

^a^Truncated gene

*fs* frame shift, *mut/Mb* mutations per megabase

**Fig. 1:**
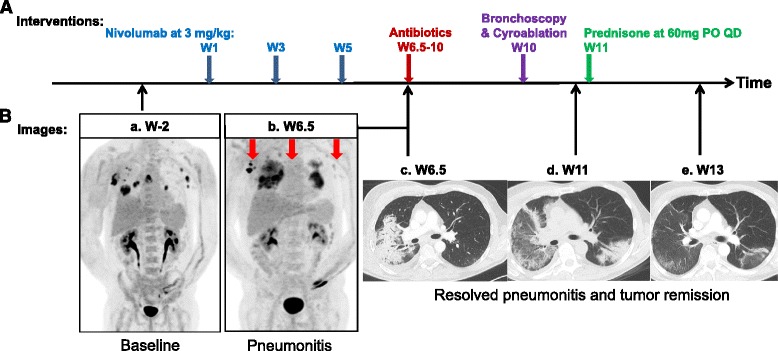
Summary of treatment and monitoring tumor response. **A** Various interventions that the patient received. *Arrowheads* indicate time points for each intervention. **B** (*a*–*e*) PET/CT or chest CT images for lung lesions before and after nivolumab. Radiologic response to nivolumab was first noted at the onset of pneumonitis as multiple ground-glass opacities surrounded by consolidations of air bronchograms in bilateral lungs on restaging PET/CT obtained at 6.5 weeks after nivolumab treatment. Subsequent CT scans showed resolution of pneumonitis and complete remission of tumor at 13 weeks

The patient received docetaxel and an investigational agent on a clinical trial for recurrent disease. Despite an initial partial response after 2 cycles of treatment, the patient had rapid tumor progression radiographically by PET/CT scan after 6 cycles of treatment. He subsequently started on standard of care nivolumab at 3 mg/kg intravenously every 2 weeks based on CheckMate-057 [9]. The patient reported increasing dyspnea on exertion (DOE) and fatigue during his clinic visit for pre-cycle 3 evaluation at week 4 day 3 (i.e., cycle 2 day 10) (Fig. 1A). He denied any productive cough, fever, or night sweats. Chest x-ray on the same day revealed no acute event although it was difficult to compare the complex lung lesions to those on the prior PET and CT scans. The patient proceeded with his third dose of nivolumab at week 5 as planned.

Due to worsening DOE, a follow-up PET/CT scan at week 6.5 was performed (Fig. 1B, b and c compared to a), revealing: (1) interval development of a moderate to large right pleural effusion with adjacent compressive atelectasis, (2) significant increase in the surrounding FDG activity in the preexisting large cavitary lesion in the RLL, and (3) increased RLL consolidation with diffuse FDG activity. It was unclear if the findings in the RLL represented pneumonia, tumor progression, or drug reaction. Interestingly, excluding these lung parenchymal findings, there was significant decrease in the FDG activity and size of known tumors in the bilateral axillary and mediastinal nodes (red arrows in Fig. 1B, b).

**Fig. 2:**
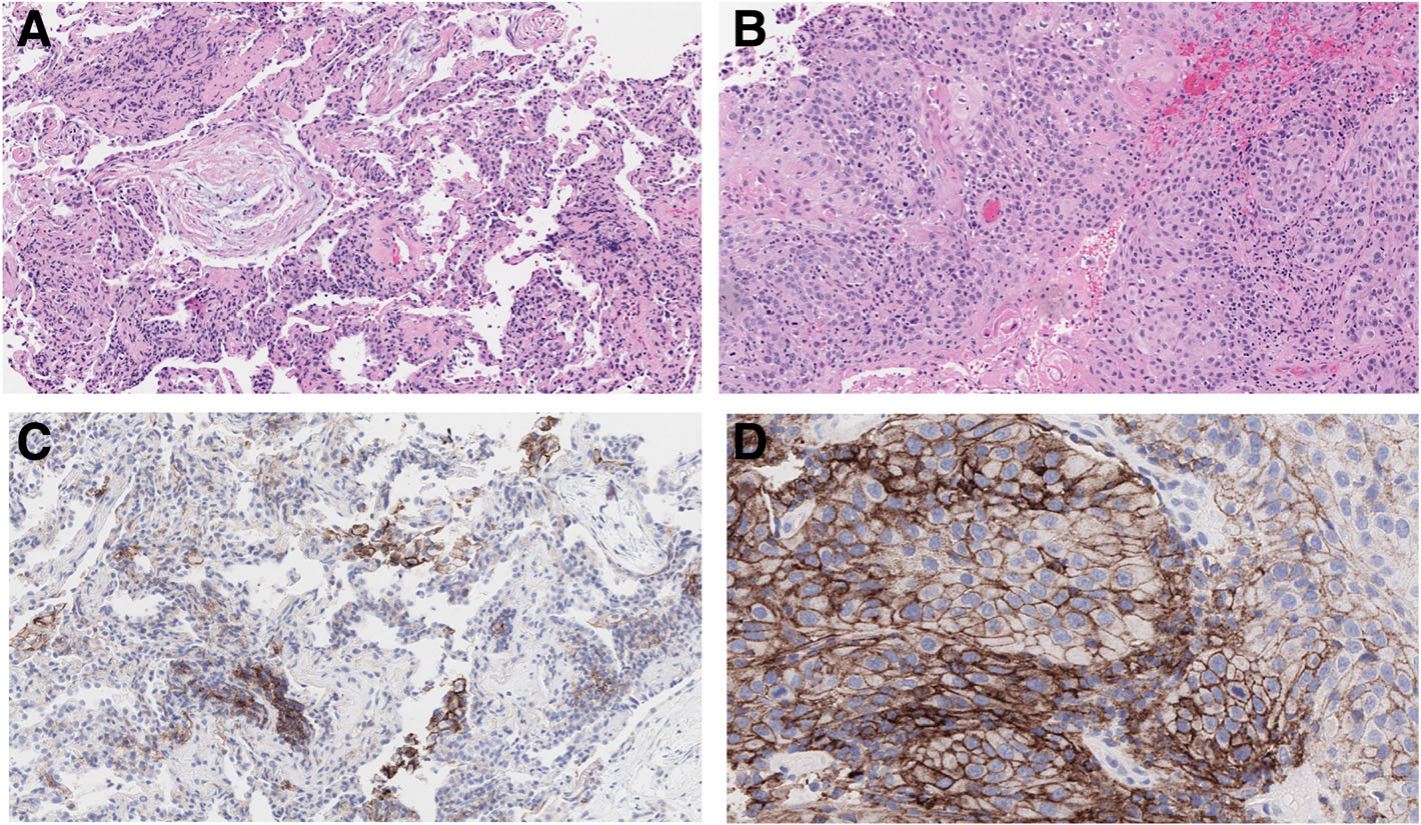
Histological assessment of different lung pathologies. Hematoxylin and eosin staining revealed organizing pneumonia with fibroblast plugs and inflammatory cells in the RML (**a**), and moderately differentiated squamous cell carcinoma in the RLL (**b**). Magnification, ×20. IHC staining for PD‐L1 expression shows the presence of PD-L1 positive intra-alveolar macrophages in the RML (**c**) and strongly positive PD-L1 expression on the surface of 90% of viable tumor cells (TPS ≥50%) (**d**). Magnification, ×100

The patient was briefly admitted for possible severe pneumonia and received empiric intravenous antibiotics. He was discharged on oral antibiotics after all cultures were negative for infection. However, the patient’s DOE and fatigue did not improve and pneumonitis developed in the LLL of the lung on chest CT scan (Fig. 1B, d). A diagnostic bronchoscopy was performed approximately 4 weeks later, which revealed no evidence of infection and two different pathologic lesions, i.e., organizing pneumonia in the right middle lobe (RML) (Fig. 2a) and extensive endobronchial, moderately differentiated SCC tumors in the RLL bronchus (Fig. 2b), which were debrided from the RLL bronchus with a cryoprobe. Although the patient had received two lines of chemotherapy (gemcitabine and paclitaxel combination and docetaxel) in the prior year with the last dose of chemotherapy that was more than 2 months ago, there was no distinct tumor necrosis or treatment effect noted. There was focal inflammation within the tumor but no distinct tumor cell reaction to the inflammation (Fig. 2a). These findings suggest freshly growing recurrent tumors in the RLL. Conversely, there was no visible tumor seen in the RML and transbronchial biopsy revealed alveolar parenchyma with fibroblast foci, mild collagen expansion of alveolar septa, and non-specific chronic inflammation (Fig. 2b). These features as well as the patient’s clinical course suggested the diagnosis of nivolumab-induced organizing pneumonia. We further determined the PD-L1 expression on these specimens by the FDA-approved IHC assay (PD-L1 22C3 IHC pharmDx, Dako—Agilent Technologies). We found PD-L1-positive cells infiltrating in the RML specimen were predominantly intra-alveolar macrophages (Fig. 2c), which may have contributed to the development of pneumonitis. There was insufficient normal lung tissue present in this specimen for determining if PD-L1 expression on normal lung tissue may also have contributed to the development of site-specific pneumonitis. In contrast, 90% of viable tumor cells in the RLL had high PD-L1 expression, i.e., tumor proportion score (TPS) ≥50% with partial and complete cell membrane staining (Fig. 2d).

Immediately after the initiation of prednisone at 60 mg per day, the patient had symptomatic improvements with decreased DOE and chest tightness and increased energy and exercise tolerance. Chest CT scan about 2 weeks later (i.e., by week 13) revealed dramatic improvement in pneumonitis (Fig. 1B, e). The patient felt that he was nearly back to baseline 3 weeks later. He had a “flare” of pneumonitis symptoms when steroids were quickly tapered off, and subsequently improved with re-initiation of steroids with a slow taper over 2 months. Despite treatment being discontinued after three doses of nivolumab, continued tumor response to a complete remission between week 5 and week 13 (i.e., ~3 months) was evident by radiographic assessment. At the last follow-up before the submission of this report, the patient has remained in clinical remission for 14 months.

**Fig. 3:**
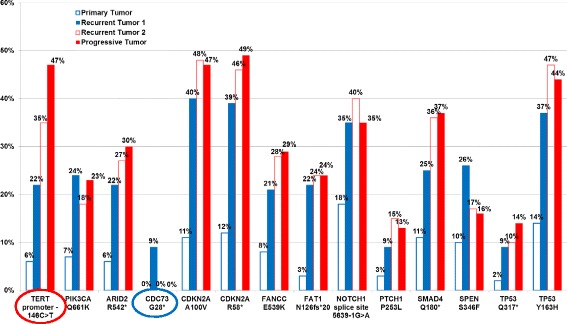
Comparison of functional genomic alterations by FoundationOne assay. Graphic comparison of MAF% for each functional genomic alteration detected by FoundationOne targeted exome sequencing assay in the initial (*column A*), recurrent and metastatic (*columns B and C*), and post-treatment, relapsed tumor (*column D*)

The complex diagnostic challenges encountered in this case lead to tumor genomic profiling analysis of four tumor specimens by FoundationOne assay obtained at initial diagnosis, tumor recurrence, and pneumonitis/tumor progression (Fig. 3 and Table 1), a clinical strategy that is not routinely employed for patients with advanced SCC with no driver mutations. We found that all tumor specimens from this patient had a very high tumor mutation burden (TMB) at 87.4–91.0 and 82.9 mut/Mb in the pre- and post-nivolumab tumor specimens, respectively, corresponding to the 95–96 percentile of TMB in lung SCC (Table 1).

## Discussion

The clinical characteristics, radiographic patterns, and treatment course of PD-1 inhibitor-related pneumonitis are quite variable [8, 10, 11]. A recent systematic review and meta-analysis suggests that the overall incidence of pneumonitis during PD-1 inhibitor monotherapy was 2.7% (95%CI, 1.9–3.6%) for all-grade and 0.8% (95%CI, 0.4–1.2%) for grade 3 or higher pneumonitis. The incidence was higher in NSCLC for all-grade (4.1 vs 1.6%; *P* = 0.002) and grade 3 or higher (1.8 vs 0.2%; *P* < 0.001) pneumonitis compared to melanoma [11]. The incidence of pneumonitis was more frequent during combination therapy than monotherapy for all-grade (6.6 vs 1.6%; *P* < 0.001) and grade 3 or higher (1.5 vs 0.2%; *P* = 0.001) pneumonitis [11]. The median time from therapy initiation to pneumonitis was 2.6 months, although it could occur after one or two doses of a PD-1/PD-L1 inhibitor [11]. Variable clinicopathological factors, such as preexisting lung damage due to tumor burden, smoking, chronic obstructive pulmonary disease, pulmonary fibrosis, and variable expression of PD-L1 on normal lung tissues, have all been associated with a higher incidence of pneumonitis in NSCLC patients compared to other tumor types. However, the implicated factors are highly variable for individual patients [10]. Our patient had grade 1 radiation-induced pneumonitis in the RUL after receiving radiation to the right axilla, which was unlikely to have contributed to the development of grade 3 pneumonitis present in the RLL and LLL. We postulated that the infiltration of PD-L1-positive macrophages played a role in the distribution and severity of pneumonitis in the RML and potentially other lung parenchymal areas. Our finding suggests macrophage-mediated inflammatory responses may have contributed to the pathogenesis of pneumonitis in this area. Early recognition and prompt initiation of high dose, systemic corticosteroids and supportive care in our patient resulted in resolution of pneumonitis without compromise of the antitumor effect. It is unlikely that different clones of PD-1+ CD8+ T cells might have mediated antitumor effect and/or irAE (pneumonitis here) [12], although we could not entirely rule out the expression of PD-L1 on normal lung epithelial cells and other immune regulators in the tumor microenvironment (TME) due to the small size of available tissue specimen obtained by transbronchial fine-needle aspiration. Further mechanistic studies on more cases are needed to fully understand the underlying mechanisms and develop clinical guideline for evaluation and management of PD-1/PD-L1 inhibitor-induced pneumonitis.

The patient’s tumor has several molecular characteristics that have been associated with a favorable clinical response to PD-1 inhibitors. First, the majority of post-nivolumab viable tumor cells stained highly positive (TPS ≥50%) for PD-L1 protein expression by IHC (Fig. 2d). Second, all tumor specimens from this patient had a very high TMB (Table 1). TMB was calculated by counting somatic genomic alterations detected per megabase of the coding region target territory of the FoundationOne test (currently 1.11 Mb for targeted exome sequencing of 315 oncogenes and tumor suppressor genes; Foundation Medicine, Cambridge, MA, USA), after filtering to remove known somatic and deleterious mutations as described previously [13, 14]. High TMB (>16 mut/Mb) by FoundationOne has been independently associated with improved PFS and OS in patients with advanced NSCLC who had received atezolizumab and other PD-1 or PD-L1 inhibitors [15, 16]. Although restricting to the FoundationOne capture regions significantly reduced the total number of genomic alterations detected, the whole exome and FoundationOne targeted exome sequencing counts were highly correlated using The Cancer Genome Atlas (TCGA) bladder urothelial carcinoma exome-sequencing database [14]. Except for one mutation (CDC73 G28*), 13 “functional” genomic alterations, which are defined as known or likely oncogenic genomic alterations, detected in the post-treatment, relapsed tumor (column D) were identical to those of the initial (column A) and recurrent metastatic (columns B and C) tumors. Of note, the mutation allele frequency (MAF) of the identified genomic mutations was usually higher in the recurrent (columns B and C) and progressive (column D) tumors than those of the initial tumor (column A) (Fig. 3). We believe the high MAFs were mainly driven by the fraction of tumor present in the biopsy specimens. These data suggest that it was unlikely that a new neoantigen or tumor-associated antigen (TAA) had emerged in the relapsed tumor compared to the primary or recurrent metastatic tumors in this patient. This phenomenon is quite different from oncogene-driven tumors where stepwise, somatic-cell mutations with sequential, subclonal selections occur during cancer progression and the development of acquired resistance to molecularly targeted therapy [17, 18]. Third, all tumors harbored a *PIK3CA* Q661K mutation and mutations in several DNA damage response genes (Table 1) that have been associated with increased clinical responses to PD-1 or PD-L1 inhibitors [19–21]. Fourth, among all the functional genomic alterations detected, telomerase reverse transcriptase (*TERT* or *hTERT*) promoter -146C>T mutation had incrementally increased in MAF% over time (Fig. 3 and Table 1), which could serve as a TAA. Several therapeutic approaches targeting TERT are under development, including immunotherapies utilizing TERT as a TAA, antisense oligonucleotide- or peptide-based therapies, and *TERT* promoter-directed cytotoxic molecules [22, 23]. We do not know if whole exome sequencing could identify additional TAA candidates. Further exploration and validation of these molecular biomarkers and potential TAA(s) is warranted.

Discontinuation of nivolumab was recommended in our patient due to the presence of grade 3 pneumonitis. Luckily, at the diagnosis of pneumonitis at 4–5 weeks after initiating nivolumab treatment, we observed radiographic responses of existing tumors (Fig. 1), suggesting the rapid activation of presumably PD-1+, tumor-specific, CD8+ T cells. These potent CD8+ T cells were able to eradicate all established and newly formed biopsy-proven tumors in the RLL by ~3 months. Thus, radiographic evaluation alone did not assess the functional status of host immunity against cancer in our patient after cancer immunotherapy. The continued and sustained antitumor response in our patient beyond a year after discontinuing nivolumab challenges the current clinical recommendation of continuing PD-L/PD-L1 treatment for tumor progression for 2 years. Currently, the patient is under radiographic surveillance every 3–4 months as standard of care for patients with metastatic NSCLC. Moving forward, a noninvasive biomarker assay that can evaluate the status of host immunity against tumor should be developed to evaluate or monitor the status of immune function in cancer patients who have responded to PD-1/PD-L1 inhibitor therapy [20].

Late tumor relapse has been reported for five advanced melanoma patients enrolled in the pivotal phase I study [24], who were retreated at their originally assigned dose and achieved durable disease control (one had a complete response) over time and were alive at 5 years [25]. Should our patient have tumor recurrence, the efficacy and safety of using nivolumab or another PD-1/PD-L1 inhibitor is unknown. There is a critical need to understand the mechanisms underlying the exceptional tumor response and treatment-related pneumonitis to guide subsequent clinical management of this patient, ideally before his tumor recurs. In this patient, rapid onset of effector T cell activation and tumor response against the preexisting tumors (red arrows in Fig. 1B, b) was noted when radiographic assessment was done for evaluating severe pneumonitis. But this immune response was not sufficient to eradicate all tumors or stop the new tumor growth in the RML at that time (Figs. 1B, b and 2b). Despite discontinuation of further treatment, radiographic tumor remission in this patient with chemotherapy-refractory, metastatic lung SCC was achieved by week 13 (i.e., ~3 months) and maintained at 14 months after the last dose of nivolumab (Fig. 1B, e). Of interest, the antitumor effect of nivolumab was also not compromised by the two lines of cytotoxic chemotherapy within 1 year immediately prior to nivolumab treatment [9, 26]. Consistent with the Chen hypothesis [27–30], nivolumab likely elicited exceptional tumor response in this patient by resetting (or “reestablishing or normalizing”) host immunity against the tumor. Further analysis of clonal expansion of T cell receptor and B cell receptor repertoires in post-nivolumab lymphocytes and identifying TAA could help to prove this assumption. To elicit cytolysis of tumor cells, effector T cells rely on tumor expression of target antigens in a human leukocyte antigen (HLA)-restricted manner that could be predicted using experimental and mathematical models [31, 32]. High resolution HLA typing revealed homozygous HLA-A*0201 allele (Table 2), which is the globally prevalent HLA class I allele that has been used to study cancer vaccines for years and recently to develop universal cancer vaccine targeting TERT-derived peptides [32].

**Table 2:** High resolution HLA typing

Haplotype	A*	B*	C*	DRB1*	DQB1*	DPB1*
	02:01	44:02	05:01	03:01	02:01	
		57:01	07:01	07:01	03:03	

Our study has several limitations. First, we did not understand the mechanisms of immune evasion in this patient. Second, we could not identify and characterize the effector immune cells, cytokines, and chemokines that are responsible for the potent and durable antitumor effect in this patient. Identifying the specific T cell receptor (TCR)(s) and HLA-restricted TAA(s) using serial tumor and blood specimens obtained before, during, and after discontinuation of nivolumab in this patient would help to understand the T cell responses that are responsible for such an exceptional antitumor response and could help design a personalized cancer vaccine or adoptive T cell therapy to improve the efficacy and safety of cancer immunotherapy should our patient have tumor recurrence. Third, we could not characterize the expression of tumor cells and immune cells in the TME in more than two areas of the lung that were responsible for the variable tumor responses and pneumonitis. Fourth, we did not determine whether or not other genetic factors, such as genetic polymorphisms in cytokine genes and pharmacogenetics, were involved in modulating the immune responses in our patient [33]. As PD-1/PD-L1 inhibitors have been rapidly integrated into standard of care for NSCLC and increasing numbers of other cancer types [34–38], more mechanistic studies are needed to understand the underlying mechanisms of tumor response and resistance. Also needed is the development of biomarkers, not only to predict treatment effects but also potentially fatal toxicities.

## Conclusions

We report our multidisciplinary clinical experience and molecular and immune biomarker studies in a patient with refractory, lung SCC who developed grade 3 pneumonitis after three doses of nivolumab monotherapy concurrent with the onset of a potent antitumor response that led to a durable clinical remission. We found that high PD-L1 expression by IHC staining was seen in intra-alveolar macrophages and viable tumor cells in the pneumonitis and recurrent tumor specimens, respectively. The prompt recognition and institution of oral steroids appeared to have prevented serious morbidity and potential mortality from pneumonitis without compromising the remarkable antitumor effect. Despite discontinuation of treatment after only three doses of nivolumab, continued tumor response to a complete remission at ~3 months evident by radiographic assessments has been maintained up to the time of submission of this report at 14-month follow-up. To the best of our knowledge, this is the first report of comprehensive tumor genomic profiling on serial tumor specimens from a lung SCC patient who has had an exceptional clinical response to nivolumab. There are critical needs (1) to delineate the mechanisms underlying exceptional tumor responses and severe tissue-specific, immune-mediated toxicities, (2) to develop noninvasive assay(s) for evaluating the status of antitumor host immunity, and (3) to develop personalized immunotherapy strategies to improve the effectiveness and safety of cancer immunotherapy.

## Abbreviations

CT: Computed tomography
DOE: Dyspnea on exertion
FDG: Fluorodeoxyglucose
HLA: Human leukocyte antigen
IHC: Immunohistochemistry
irAEs: Immune-related adverse effects
LLL: Left lower lobe
MAF: Mutation allele frequency
Mut/Mb: Mutations per megabase
NSCLC: Non-small-cell lung cancer
PD-1: Programmed cell death-1
PD-L1: Programmed cell death 1 ligand 1
PET: Positron emission tomography
RLL: Right lower lobe
RML: Right middle lobe
RUL: Right upper lobe
SCC: Squamous cell carcinoma
SUVmax: Maximum standardized uptake values
TAA: Tumor-associated antigen
TCGA: The Cancer Genome Atlas
TCR: T cell receptor
TERT: Telomerase reverse transcriptase (or hTERT)
TMB: Tumor mutation burden
TME: Tumor microenvironment
TPS: Tumor proportion score

## References

[1] Brahmer J, Reckamp KL, Baas P, Crino L, Eberhardt WE, Poddubskaya E (2015). Nivolumab versus docetaxel in advanced squamous-cell non-small-cell lung cancer. N Engl J Med.

[2] Herbst RS, Baas P, Kim DW, Felip E, Perez-Gracia JL, Han JY (2016). Pembrolizumab versus docetaxel for previously treated, PD-L1-positive, advanced non-small-cell lung cancer (KEYNOTE-010): a randomised controlled trial. Lancet.

[3] Fehrenbacher L, Spira A, Ballinger M, Kowanetz M, Vansteenkiste J, Mazieres J (2016). Atezolizumab versus docetaxel for patients with previously treated non-small-cell lung cancer (POPLAR): a multicentre, open-label, phase 2 randomised controlled trial. Lancet.

[4] Rittmeyer A, Barlesi F, Waterkamp D, Park K, Ciardiello F, von Pawel J (2016). Atezolizumab versus docetaxel in patients with previously treated non-small-cell lung cancer (OAK): a phase 3, open-label, multicentre randomised controlled trial. Lancet.

[5] Reck M, Rodriguez-Abreu D, Robinson AG, Hui R, Csoszi T, Fulop A (2016). Pembrolizumab versus chemotherapy for PD-L1-positive non-small-cell lung cancer. N Engl J Med.

[6] Antonia S, Goldberg SB, Balmanoukian A, Chaft JE, Sanborn RE, Gupta A (2016). Safety and antitumour activity of durvalumab plus tremelimumab in non-small cell lung cancer: a multicentre, phase 1b study. Lancet Oncol.

[7] Nishino M, Sholl LM, Hodi FS, Hatabu H, Ramaiya NH (2015). Anti-PD-1-related pneumonitis during cancer immunotherapy. N Engl J Med.

[8] Nishino M, Ramaiya NH, Awad MM, Sholl LM, Maattala JA, Taibi M (2016). PD-1 inhibitor-related pneumonitis in advanced cancer patients: radiographic patterns and clinical course. Clin Cancer Res.

[9] Borghaei H, Paz-Ares L, Horn L, Spigel DR, Steins M, Ready NE (2015). Nivolumab versus docetaxel in advanced nonsquamous non-small-cell lung cancer. N Engl J Med.

[10] Naidoo J, Wang X, Woo KM, Iyriboz T, Halpenny D, Cunningham J (2016). Pneumonitis in patients treated with anti-programmed death-1/programmed death ligand 1 therapy. J Clin Oncol.

[11] Nishino M, Giobbie-Hurder A, Hatabu H, Ramaiya NH, Hodi FS (2016). Incidence of programmed cell death 1 inhibitor-related pneumonitis in patients with advanced cancer: a systematic review and meta-analysis. JAMA Oncol.

[12] Uemura M, Trinh VA, Haymaker C, Jackson N, Kim DW, Allison JP (2016). Selective inhibition of autoimmune exacerbation while preserving the anti-tumor clinical benefit using IL-6 blockade in a patient with advanced melanoma and Crohn’s disease: a case report. J Hematol Oncol.

[13] Frampton GM, Fichtenholtz A, Otto GA, Wang K, Downing SR, He J (2013). Development and validation of a clinical cancer genomic profiling test based on massively parallel DNA sequencing. Nat Biotechnol.

[14] Rosenberg JE, Hoffman-Censits J, Powles T, van der Heijden MS, Balar AV, Necchi A (2016). Atezolizumab in patients with locally advanced and metastatic urothelial carcinoma who have progressed following treatment with platinum-based chemotherapy: a single-arm, multicentre, phase 2 trial. Lancet.

[15] Spigel DR, Schrock AB, Fabrizio D, Frampton GM, Sun J, He J (2016). Total mutation burden (TMB) in lung cancer (LC) and relationship with response to PD-1/PD-L1 targeted therapies. J Clin Oncol.

[16] Kowanetz M, Zou W, Shames DS, Cummings C, Rizvi N, Spira AI (2016). Tumor mutation load assessed by FoundationOne (FM1) is associated with improved efficacy of atezolizumab (atezo) in patients with advanced NSCLC. Ann Oncol.

[17] Greaves M, Maley CC (2012). Clonal evolution in cancer. Nature.

[18] Vogelstein B, Papadopoulos N, Velculescu VE, Zhou S, Diaz LA, Kinzler KW (2013). Cancer genome landscapes. Science.

[19] Le DT, Uram JN, Wang H, Bartlett BR, Kemberling H, Eyring AD (2015). PD-1 blockade in tumors with mismatch-repair deficiency. N Engl J Med.

[20] Ma W, Gilligan BM, Yuan J, Li T (2016). Current status and perspectives in translational biomarker research for PD-1/PD-L1 immune checkpoint blockade therapy. J Hematol Oncol.

[21] Chen KH, Yuan CT, Tseng LH, Shun CT, Yeh KH (2016). Case report: mismatch repair proficiency and microsatellite stability in gastric cancer may not predict programmed death-1 blockade resistance. J Hematol Oncol.

[22] Akincilar SC, Khattar E, Boon PL, Unal B, Fullwood MJ, Tergaonkar V (2016). Long-range chromatin interactions drive mutant TERT promoter activation. Cancer Discov.

[23] Yuan P, Cao JL, Abuduwufuer A, Wang LM, Yuan XS, Lv W (2016). Clinical characteristics and prognostic significance of TERT promoter mutations in cancer: a cohort study and a meta-analysis. PLoS One.

[24] Topalian SL, Sznol M, McDermott DF, Kluger HM, Carvajal RD, Sharfman WH (2014). Survival, durable tumor remission, and long-term safety in patients with advanced melanoma receiving nivolumab. J Clin Oncol.

[25] Hodi FS, Kluger H, Sznol M, Carvajal R, Lawrence D, Atkins M (2016). Abstract CT001: durable, long-term survival in previously treated patients with advanced melanoma (MEL) who received nivolumab (NIVO) monotherapy in a phase I trial. Cancer Res.

[26] Kazandjian D, Suzman DL, Blumenthal G, Mushti S, He K, Libeg M (2016). FDA approval summary: nivolumab for the treatment of metastatic non-small cell lung cancer with progression on or after platinum-based chemotherapy. Oncologist.

[27] Chen L, Han X (2015). Anti-PD-1/PD-L1 therapy of human cancer: past, present, and future. J Clin Invest.

[28] Sanmamed MF, Chen L (2014). Inducible expression of B7-H1 (PD-L1) and its selective role in tumor site immune modulation. Cancer J.

[29] Zhang Y, Chen L (2016). Classification of advanced human cancers based on tumor immunity in the MicroEnvironment (TIME) for cancer immunotherapy. JAMA Oncol.

[30] Wang J, Yuan R, Song W, Sun J, Liu D, Li Z (2017). PD-1, PD-L1 (B7-H1) and tumor-site immune modulation therapy: the historical perspective. J Hematol Oncol.

[31] Kalaora S, Barnea E, Merhavi-Shoham E, Qutob N, Teer JK, Shimony N (2016). Use of HLA peptidomics and whole exome sequencing to identify human immunogenic neo-antigens. Oncotarget.

[32] Robbins PF, Lu YC, El-Gamil M, Li YF, Gross C, Gartner J (2013). Mining exomic sequencing data to identify mutated antigens recognized by adoptively transferred tumor-reactive T cells. Nat Med.

[33] Pandey GS, Sauna ZE (2014). Pharmacogenetics and the immunogenicity of protein therapeutics. J Interferon Cytokine Res.

[34] Hirsch FR, Suda K, Wiens J, Bunn PA (2016). New and emerging targeted treatments in advanced non-small-cell lung cancer. Lancet.

[35] Dholaria B, Hammond W, Shreders A, Lou Y (2016). Emerging therapeutic agents for lung cancer. J Hematol Oncol.

[36] Hsueh EC, Gorantla KC (2015). Novel melanoma therapy. Exp Hematol Oncol.

[37] McCaughan GJ, Fulham MJ, Mahar A, Soper J, Hong AM, Stalley PD (2016). Programmed cell death-1 blockade in recurrent disseminated Ewing sarcoma. J Hematol Oncol.

[38] Alexander GS, Palmer JD, Tuluc M, Lin J, Dicker AP, Bar-Ad V (2016). Immune biomarkers of treatment failure for a patient on a phase I clinical trial of pembrolizumab plus radiotherapy. J Hematol Oncol.

